# The Role of p16/Ki67 Dual Staining in Cervical Cancer Screening

**DOI:** 10.3390/cimb45100534

**Published:** 2023-10-19

**Authors:** Andraž Dovnik, Alenka Repše Fokter

**Affiliations:** 1 University Clinic for Gynaecology and Obstetrics, University Medical Centre Maribor, Ljubljanska 5, 2000 Maribor, Slovenia; 2 Department of Pathology and Cytology, General Hospital Celje, Oblakova 5, 3000 Celje, Slovenia; alenka.repse-fokter@guest.arnes.si

**Keywords:** cervical intraepithelial lesion, human papillomavirus, cervical cancer screening

## Abstract

Cervical cancer screening has enabled a decrease in the incidence and mortality of cervical cancer. Various screening modalities have been studied to date. In many countries, screening is still based on cervical cytology, where cervical cells obtained either on glass or in a liquid medium are examined under a microscope. However, the fact that the vast majority of cervical cancers are a result of persistent infection with high-risk human papillomaviruses (hr-HPV) has led to the implementation of primary HPV screening in many countries. Taking into consideration the fact that the majority of HPV infections are transient and do not cause cervical precancer, effective triage methods are needed to prevent an increase in colposcopy referrals. Among these, the most extensively investigated are HPV genotyping, HPV methylation, and p16/Ki67 dual staining. In this manuscript, we briefly summarize the current knowledge regarding different screening strategies for the prevention of cervical cancer, with a focus on p16/Ki67 dual staining. In addition, we provide an explanation regarding the rationale for the use of various screening modalities based on the molecular biology of cervical cancer and cervical precancerous lesions.

## 1. Introduction

For many decades, cervical cancer screening was based on cervical cytology [1]. This can be performed either by conventional cytology (CC) or by liquid-based cytology (LBC) [2]. However, this method has relatively low sensitivity due to the high rate of false-negative results [3,4,5]. To increase its effectiveness, the test should be frequently repeated. In addition, it requires highly trained personnel and complex infrastructure [1]. As a result of this drawback and the fact that the majority of cervical cancers develop as a consequence of persistent infection with high-risk human papillomavirus (hr-HPV), many countries have implemented primary HPV screening [1,6]. However, the majority of HPV infections are productive, clear spontaneously, and do not cause cervical dysplasia, so HPV screening has high rates of false-positive results. This in turn leads to lower specificity, especially in women younger than 30 years [1].

To decrease the number of unnecessary colposcopy referrals, an appropriate triage test for HPV-positive women is needed. In the majority of countries, cervical cytology is used as a triage test [1]. However, many other triaging methods have been extensively investigated, such as p16/Ki67 dual staining, HPV genotyping, and HPV methylation tests.

We performed a systematic review of the literature on the role of p16/Ki67 dual staining in cervical cancer screening. The literature search was conducted using the MEDLINE electronic database. After screening for relevant content among the 120 identified papers, we selected 102 papers until September 2023. Peer-reviewed articles published in the English language and containing an abstract were considered, and reference lists were screened for additional relevant citations. Some additional manuscripts were added due to their close association with the content of the manuscript. Full-text versions of all manuscripts were obtained. The systematic review was conducted in accordance with PRISMA guidelines.

In this review, we briefly summarize the current knowledge regarding the most commonly used screening techniques for cervical cancer. In the first part, we focus on the studies that evaluated the effectiveness of cytology and HPV testing in the prevention of cervical cancer. In the second part of the manuscript, we describe in depth the molecular biology of cervical cancer development as a result of hr-HPV infection. In the final part, we summarize current knowledge regarding p16/Ki67 dual staining and propose further research regarding the use of p16/Ki67 dual staining in cervical cancer screening.

## 2. Conventional and Liquid-Based Cytology

Cervical cancer screening based on CC by the method of Papanicolaou (PAP) significantly decreased the incidence and mortality of cervical cancer [7]. The smear of the ectocervix is obtained with an Ayre spatula, and the smear of the endocervix is obtained with a cytobrush. The smear should be obtained without menstrual bleeding or local therapy, and it has to be immediately fixed to the glass. In terms of specimen quality, the smears are considered satisfactory, satisfactory but limited, and unsatisfactory when cellular debris clouding together with blood and inflammation are present in more than 75% of the smear [2].

**Figure 1:**
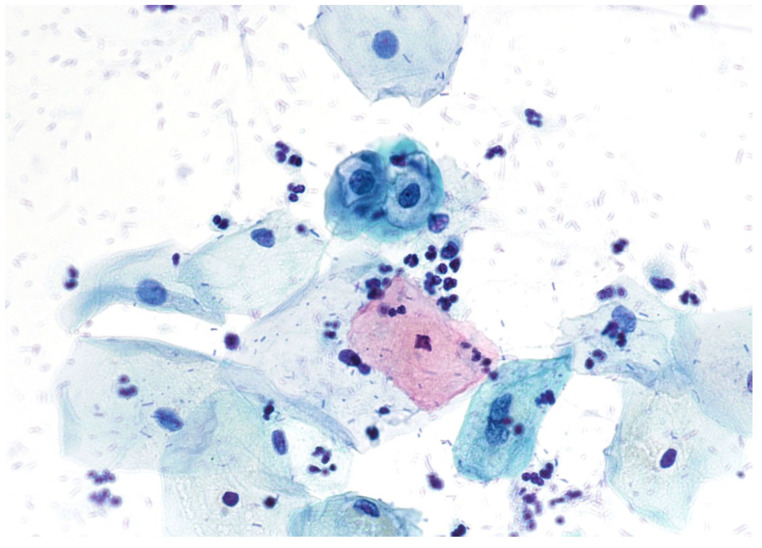
LSIL Papanicolaou stain, ×400.

Cervical smears are classified according to the Bethesda system [8]. Abnormalities of squamous and glandular cells can be detected on cervical smears [2]. The most common type of abnormal cervical smear is ‘atypical squamous cells of unknown significance’ (ASC-US), which accounts for about 5% of all smears in the normal population [9,10]. However, this result is associated with cervical intraepithelial neoplasia grade 2 or more (CIN2+) in only 9% [2,11]. ASC-US suggests the possibility of a low-grade squamous intraepithelial lesion that has not yet been confirmed by biopsy. When there is high suspicion of a high-risk squamous intraepithelial lesion that has not yet been confirmed with biopsy, the proposed smear result is ‘atypical squamous cells not permitting exclusion of a high-grade intraepithelial lesion’ (ASC-H) [2]. Another two categories suggesting a squamous lesion within the Bethesda system are LSIL (‘low grade squamous intraepithelial lesion’) and HSIL (‘high grade squamous intraepithelial lesion’). LSIL suggests productive infection with hr-HPV (Figure 1).

**Figure 2:**
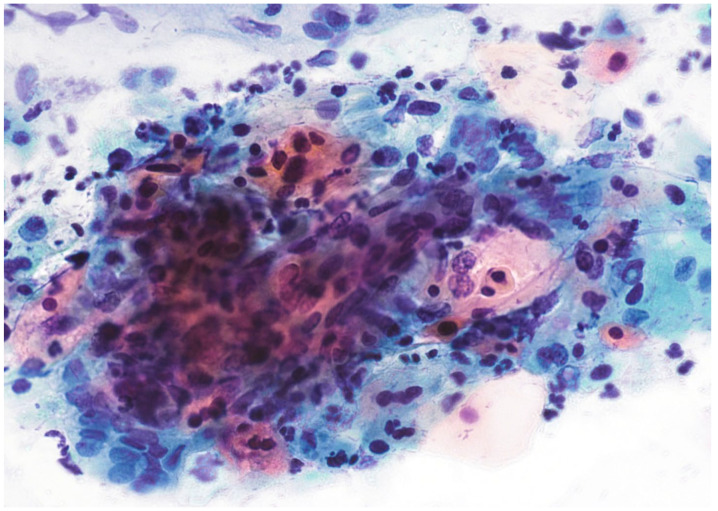
HSIL, Papanicolaou stain, ×400.

The main characteristic of this lesion is the presence of koilocytes. LSIL lesions often spontaneously regress. HSIL (Figure 2), on the other hand, is a sign of transforming HPV infection and is usually associated with biopsy-proven high-grade lesions.

**Figure 3:**
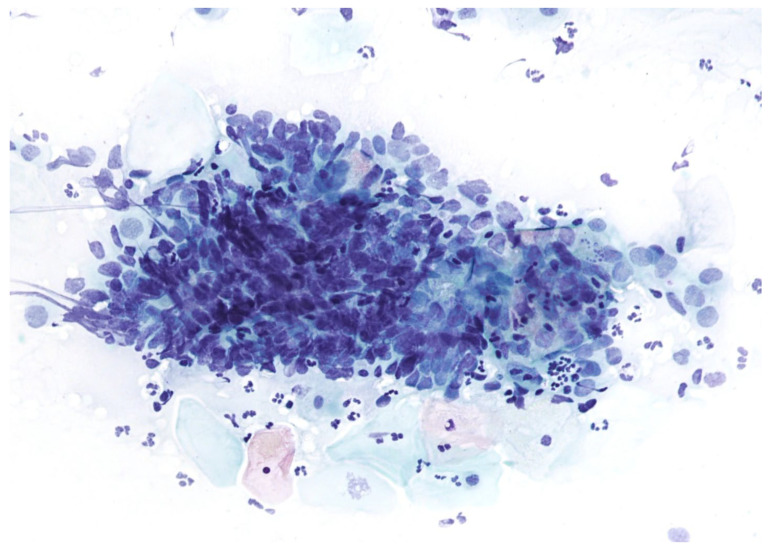
Adenocarcinoma in situ, Papanicolaou stain, ×200.

The abnormalities of glandular cells include ‘atypical glandular cells’ (AGC), endocervical adenocarcinoma in situ (AIS) (Figure 3), and adenocarcinoma.

The sampling of glandular cells may be difficult due to the often multifocal nature of AIS and the possible occurrence of the lesion in the base of the endocervical crypts, which can preclude the detection of glandular lesions [2].

The disadvantage of cervical cytology is its low sensitivity, which is in the range of 20–70%, and its high interobserver variability in the interpretation of cervical smears [12,13,14]. The diagnostic performance of cervical cytology has been studied in three big population-based studies (ATHENA (Addressing THE Need for Advanced HPV diagnostics), VUSA (VUSA-Screen study: Vrije Universiteit Medical Centre-Saltro laboratory population-based cervical screening), and POBASCAM (population-based screening trial Amsterdam)) with 25,658–47,208 participants [3,4,5]. The sensitivity for cervical intraepithelial neoplasia grade 3 or more (CIN3+) was 52.8–75.4% and the specificity was 78.0–85.6% [3,4,5]. Hence, this investigation requires an experienced observer [15,16].

The latest systematic review published in the Cochrane library included 61,099 women with ASC-US+ [17]. The sensitivity of PAP ASC-US+ (atypical squamous cells of unknown significance or higher abnormalities) for CIN2+ was 43–96% and for CIN3+ was 39–85%. The specificity for CIN2+ was 86–98% and for CIN3+ was 85–98%. In the same systematic review, LSIL+ (low-grade intraepithelial lesions or higher abnormalities) on conventional smears were analyzed in 41,494 women. The sensitivity for CIN2+ was 18–89% and for CIN3+ 64–80%, whereas the specificity for both entities was 95–98% [17].

The alternative to Pap smears is LBC, which has, in many countries, replaced CC as it offers good cost benefits due to the lower rate of inadequate smears (in the range of 1% compared to 7% in conventional cytology) [2,13,18]. This method also allows the possible automated reading of smears [2]. In addition, LBC can be used for subsequent molecular analysis [19]. The sample is obtained using the brush or a combination of brush and spatula and then rinsed in a liquid fixative [2]. The previously mentioned meta-analysis additionally analyzed the diagnostic performance of ASC-US+ and LSIL+ on liquid-based cytology [17]. The sensitivity and specificity of ASC-US+ were 52–94% and 73–97% for the detection of CIN2+ and 52–98% and 73–97% for the detection of CIN3+, respectively. The sensitivity and specificity of LSIL+ were 42–87% and 90–99% for the detection of CIN2+ and 48–93% and 92–98% for the detection of CIN3+, respectively [17].

These two methods have been compared in many comparative studies that provided conflicting results [20,21,22,23,24,25,26,27]. In 2008, Arbyn et al. published a meta-analysis of eight studies and reported no difference between CC and LBC in terms of sensitivity and specificity for the detection of high-risk lesions [19]. A Dutch randomized trial that included 89,784 women showed no difference in the diagnostic performance between the two methods [28]. Another Dutch comparative study included two cohorts with more than 86,000 participants [20]. The authors reported improved sensitivity in terms of histologically proven lesion detection with no statistical difference in specificity [20]. A Korean comparative study included 13,299 cases of CC and 15,591 cases of LBC [29]. The rate of unsatisfactory results was lower in the LBC group compared to the CC group [29]. A subsequent Japanese meta-analysis of 13 comparative studies found no statistical difference in terms of unsatisfactory smear results [30]. A German comparative study compared 11,331 LBC cases and 9296 cases of CC in women attending cervical cancer screening [31]. The relative sensitivity of LBC compared to CC was higher for the detection of CIN1+, CIN2+, and CIN3+ [31]. A Japanese comparative study evaluated the diagnostic performance of LBC compared to CC in 312 patients with ASC-US and reported no difference in the diagnostic performance [32]. Singh et al. reported a higher diagnostic accuracy of LBC compared to CC for the detection of cervical cancer recurrence in a study on 94 cervical cancer patients [33].

## 3. High-Risk HPV Test, HPV Genotyping, and HPV Methylation

The majority of cervical cancers develop after an infection with hr-HPV, and therefore testing for the presence of hr-HPV has been incorporated into the screening protocols of many countries [6,34]. Primary hr-HPV testing has higher sensitivity compared to cytology, and a negative-HPV result is more reliable in predicting the absence of a cervical premalignant lesion compared to cytology [35,36,37,38,39,40]. An assessment of the utility of primary HPV screening was made within the ATHENA study, with a follow-up analysis after three years [39]. Three screening strategies were compared (primary HPV screening, co-testing with HPV and cytology, and cytology alone). The cumulative incidence rate of CIN3+ in the cytology-negative group after three years was more than two times bigger compared to the HPV-negative group, indicating that the negative-HPV test offers better protection compared to the negative-cytology result [39]. It has also been shown that the screening interval between two HPV tests could be extended to 5 years [40,41,42]. The cohort from the POBASCAM trial was followed for 14 years. The incidence of CIN3+ and cervical cancer in the HPV-negative group after three screening rounds was similar to the incidence in the cytology-negative group after two screening rounds [41]. On the other hand, the specificity of hr-HPV testing is lower compared to cytology, and due to higher rates of false-positive results, primary HPV screening should not be used in women younger than 30 years [1].

There are many commercially available assays for HPV testing. However, only tests that show a consistently low false-negative result rate for the detection of CIN2+ or CIN3+ and at the same time detect minimal rates of transient, clinically irrelevant infections should be used in clinical practice [1]. Hr-HPV infection can be proven with the identification of viral DNA or with mRNA testing [18]. The most commonly used HPV DNA tests are HC2 (Hybrid Capture 2, Qiagen, Hilden, Germany) and the Cervista HPV HR test (Hologic, Marlborough, MA, USA). The HC2 identifies 13 HPV types (16, 18, 31, 33, 35, 39, 45, 51, 52, 56, 58, 59, and 68), and the Cervista test also identifies HPV 66. The most commonly used test based on target amplification is the Cobas 4800 test HPV (Roche Diagnostics, Tokyo, Japan), and the only mRNA test approved by the FDA is the APTIMA test [18].

To summarize, HPV testing offers high sensitivity and lower specificity for the detection of cervical precancerous lesions. If screening was based solely on HPV testing, this would result in high rates of colposcopy referrals. Therefore, many screening strategies have been studied to date to overcome this limitation, such as HPV genotyping, HPV methylation, and p16/Ki67 dual staining.

About 40 HPV genotypes, among more than 200 identified, are able to infect the cervix. Among these, HPV 16, HPV 18, and HPV 45 account for the majority of cases [43]. HPV 45 is responsible for about 5% of cervical cancer cases, HPV 18 for about 15%, and the most carcinogenic subtype, HPV 16, accounts for approximately 60% of cervical cancer cases [43]. The majority of HPV infections clear within two years, and only a small fraction persist and represent a risk factor for the development of high-risk cervical intraepithelial lesions or cervical cancer [18,43]. The utility of HPV 16/18 genotyping as a triage test for HPV-positive women compared to LBC has been studied in the ATHENA study, which included 40,901 women aged 25 or more [3]. Among women who tested positive for hr-HPV, HPV genotyping for the identification of HPV 16/18 had a similar positive-predictive value and sensitivity compared to ASC-US or more on LBC for the detection of CIN3+ [3]. Within the FOCAL randomized controlled trial on 6172 women, HPV screening with LBC triage was compared to LBC screening with HPV triage [44]. HPV testing was conducted with the HC2 test, and all HPV-positive tests were sent for genotyping. Women with normal cytology underwent repeat co-testing with LBC and hr-HPV, while women with positive baseline HPV and abnormal cytology were immediately referred to colposcopy. The study showed that 17% of women with CIN2 or more had HPV 16 or HPV 18 at baseline, which highlights the need for immediate referral to colposcopy with these genotypes identified [44]. In a recent Canadian retrospective study, genotyping was compared to cytology as a triage test for women who tested positive for hr-HPV on a sample of 1396 HPV-positive women [45]. The positive-predictive value (PPV) for the detection of CIN2+ in the first year of follow-up was calculated. The PPV of the cytology result ASC-US or more was 20.9%, compared to 31.8% for HPV 16 positive cases and 30.8% for HPV 16 or HPV 18 positive cases [45].

As a result of persistent infection with hr-HPV, many epigenetic changes occur in both viral and host DNA. The expression of these genes is changed without a change in the DNA sequence [6,46]. DNA methylation is one of the most extensively investigated epigenetic changes that occur in HPV-related cancers [47]. We recently published a review of the literature regarding the use of methylation markers in cervical cancer screening, with a specific focus on CIN2 [48]. Among the most extensively studied genes affected by HPV-induced methylation are host genes such as FAM19A4, miR124, CADM1, MAL, and PAX1 [49,50]. In a Dutch retrospective study, 1040 HPV-positive women aged 30 years or more were analyzed, and the results indicated that a negative-methylation test for FAM19A4/mir124-2 was associated with a low risk of cervical cancer development in the 4 years of follow-up [51]. A large European multicenter study analyzed the diagnostic performance of FAM19A4/mir124-2 on 2384 HPV-positive women for the detection of CIN2+ during the follow-up of two years [52]. The sensitivity for the detection of CIN3 was 77.2%, and for the detection of CIN2, it was 46.8%. The overall specificity of this test was 78.3% [52]. A methylation assay including two tumor-suppressor genes, CADM1/MAL, has also been compared to cytology and has shown comparable diagnostic performance for the detection of high-grade cervical lesions in HPV-positive women [53]. For this methylation assay, it has also been shown that the levels of methylation correspond to the duration of the hr-HPV infection and to the severity of the cervical lesion [53,54,55]. Other methylation markers that have been studied include POU Class 4 Homeobox 3 (POU4F3), paired box gene 1 (PAX1), and a methylation panel including ASTN1, DLX1, ITGA4, RXFP3, SOX17, and ZNF671 [56,57,58,59]. The methylation levels of various HPV genes have also been studied. Comparable specificity and higher sensitivity for the detection of CIN2+ compared to partial HPV 16/18 genotyping have been shown for the methylation of HPV DNA. In addition, elevated levels of methylation of L1 and L2 for HPV 16/18/33/35 in CIN3/AIS have been shown compared to normal histology [60,61,62,63]. The S5 classifier, which combines an analysis of host and HPV methylation levels (host gene EPB41L3 and genes of HPV 16, HPV 18, HPV 31, and HPV 33), has also been studied in population-based screening studies and in women referred for colposcopy [64,65,66,67]. The potential for decreasing colposcopy referrals by 50% has been shown [66]. In addition, the S5 classifier was shown to have higher sensitivity and similar specificity for the detection of CIN2+ compared to HPV 16/18 genotyping [67]. In a Finnish prospective observational study of 149 women, the S5 classifier was compared to HPV 16/18/31/33 genotyping in predicting CIN2 progression during conservative management. The S5 classifier performed better in the prediction of progression or regression [68]. Dutch researchers reported the role of FAM19A4/miR124-2 methylation in the conservative management of CIN2/3. Statistically significantly higher levels of CIN2+ spontaneous regression were shown for the negative-methylation result at the beginning of the study compared to the positive result [69].

## 4. Molecular Biology of Cervical Precancerous Lesions and Cervical Cancer

The vast majority of cervical cancers are HPV-positive. However, less than 5% of all HPV infections progress to CIN3 lesions [63,70]. HPV is a double-stranded DNA virus that interacts with host DNA. Viral DNA is organized into eight reading frames, which encode viral proteins. These include six early proteins, E1, E2, E4, E5, E6, and E7, and two late proteins, L1 and L2. Proteins E1 and E2 are involved in mechanisms of viral replication, while E6 and E7 are involved in interactions with cellular tumor suppressor genes [6,71]. The late proteins L1 and L2 form the viral capsid [71]. HPV infects the basal layer of cells in the cervical transformation zone [72]. After infection of the basal layer of the epithelium, viral DNA is kept in episomes, where gene expression is regulated [73]. The viral genome is amplified as a result of E1 and E2 expression. Viral DNA can remain in the shape of episomes for long periods of time. After many cellular divisions, the viral genome can integrate into the host genome [73]. In this case, the virus enters the nucleus and integrates into the host DNA. This integration occurs in the region of gene E2 and causes the loss of transcriptional control of E6 and E7 through their interaction with tumor-suppressor proteins [70]. E6 encodes a small zinc-binding protein that interacts with p53 and causes its degradation via a ubiquitin-dependent pathway. The amount of p53 in the affected cell is therefore reduced. p53 is one of the most important tumor-suppressor genes and is involved in the process of apoptosis. The decreased amount of p53 leads to cell cycle deregulation [70]. Normal progression through phases of the cell cycle is regulated by cyclin-dependent kinase (CDK) inhibitors. The loss of p53 function causes inhibition of CDK inhibitors by the functions of p27 and p21, and this leads to uncontrollable division of cells [74]. On the other hand, another small zinc-binding protein, E7, directly interacts with the retinoblastoma protein pRb. pRb is a tumor-suppressor protein that binds E2F, a transcription factor transcribing S-phase proteins such as CDK 4/6 inhibitors, cyclin E, cyclin A, and p16 [74]. The binding of E7 to pRb causes degradation of pRb and consequential release of E2F, which in turn leads to uncontrolled cell division [70].

## 5. The Rationale and Use of p16/Ki67 Dual Staining in Cervical Cancer Screening

**Figure 4:**
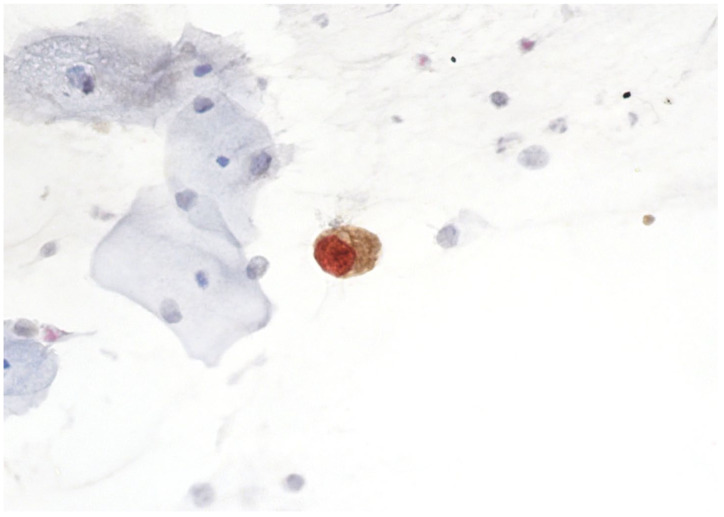
HSIL, p16/Ki67-positive reaction ×400.

p16 is a regulator of the normal cell cycle and functions as a CDK inhibitor. Its expression is increased in squamous epithelium through the E7/pRb pathway [18]. As previously mentioned, E7-mediated degradation of Rb leads to uncontrolled cellular division as a result of E2F release. p16 acts as a tumor-suppressor protein, attempting to slow the progression of the cell cycle from the G1 to the S phase through the inhibition of CDK 4/6 and the prevention of Rb phosphorylation. In cells with dysfunctional Rb, the main aim of increased p16 expression is to slow down cell cycle progression [73]. This occurs due to hr-HPV infection of these cells. The accumulation of p16 in the cytoplasm and nucleus can be identified through immunostaining. Nevertheless, p16 may be identified not only in dysplastic cells but also in normal glandular cells of the endocervical canal with squamous metaplasia [18]. On the other hand, Ki-67 is a cellular proliferation marker. p16 and Ki-67 are mutually exclusive in the normal cell, and concurrent staining of these markers in a single cell indicates oncogenic transformation as a result of hr-HPV infection [75] (Figure 4).

A large prospective study including 27,349 women who attended routine cervical cancer screening was conducted in five European countries [76]. All women underwent Pap cytology, HPV testing, and p16/Ki67 dual staining cytology. The women who had ASC-US or more on Pap cytology, positive-p16/Ki67 dual staining, and/or a positive-HPV test were referred for colposcopy, and the diagnostic performance of these tests for the detection of CIN2+ on colposcopy was calculated. The sensitivity of dual staining was higher than the Pap test, and its specificity was comparable. On the other hand, the sensitivity of dual staining was lower than HPV testing in women younger than 30 years. The results of this study also indicated that the number of false-positive results with dual staining was 50% lower compared to HPV testing, indicating that this test offers better triage for colposcopy [76]. An American study compared the diagnostic performances of dual staining and Pap cytology on hr-HPV-positive women and came to similar conclusions in terms of sensitivity and specificity for the detection of CIN2+ [77]. A subset analysis of ASC-US and LSIL cases within this large cohort was performed subsequently [78]. The sensitivity of HC2 HPV testing was comparable to p16/Ki67 dual staining in the ASC-US group for the detection of CIN2+. The specificity of dual staining in the ASC-US and LSIL groups for the detection of CIN2+ was higher compared to HC2 HPV testing [78].

A retrospective study analyzed the diagnostic performance of p16/Ki67 dual staining on a sample of 776 women with ASC-US or LSIL cytology results [75]. The results were correlated to histology results obtained during follow-up. The sensitivity of dual staining for the detection of CIN2+ was 94.2% in the LSIL group and 92.2% in the ASC-US group. The specificities in the LSIL and ASC-US groups were 68.0% and 80.6%, respectively [75]. These results were similar for groups of patients younger and older than 30 years. In both cytology groups (ASC-US and LSIL), dual staining has comparable sensitivity to HPV testing but higher specificity. These results indicated the ability of this test to decrease the number of colposcopy referrals in the ASC-US and LSIL populations [75].

A German study investigated the clinical utility of p16/Ki67 dual staining in a Pap-negative/HPV-positive population [79]. This was a sub-study of the Wolfsburg HPV screening pilot, with 425 women included. p16/Ki67 analysis was performed on stored cytology samples. The high sensitivity and specificity of p16/Ki67 dual staining for the detection of CIN2+ and CIN3+ were reported. The authors concluded that this test might be a useful adjunct for the triage of Pap-negative/HPV-positive populations [79]. An American study on 625 women referred to colposcopy evaluated the diagnostic performance of p16/Ki67 dual staining [80]. The authors reported high sensitivity rates for the detection of CIN2 and CIN3 and concluded that this test has the ability to decrease colposcopy referral rates by almost 50% [80]. Another retrospective study conducted in Denmark analyzed 469 women with LSIL cytology with minimally 5 years of follow-up [81]. High sensitivities for the detection of CIN2+ and CIN3+ were reported. In the group of patients younger than 30 years, the specificity of dual staining was higher than that of HPV testing [81]. A prospective study on 515 women compared the diagnostic performance of p16/Ki67 dual staining of ASC-US/LSIL/HSIL/ASC-H cytology to hr-HPV testing for the detection of CIN2/3 [15]. Dual staining demonstrated higher specificity and comparable sensitivity compared to hr-HPV testing [15]. The sensitivity and specificity values of DS for the detection of CIN2+ and CIN3+ from major studies performed to date are presented in Table 1.

A Dutch retrospective analysis of 847 women with Pap-negative/HPV-positive results reported a 3-year sensitivity of 73.3% and a specificity of 70.0% for the detection of CIN3+ with dual staining. The reported 5-year cumulative incidence rate for the detection of CIN3+ was 6.9% in women with normal cytology and positive-HPV, and this percentage fell to 3.3% if these women were tested with p16/Ki67 dual staining, provided the test was negative [82]. These findings were later confirmed by a large prospective cohort study of 1549 HPV-positive women [83]. Women with negative dual staining had a lower 5-year risk of developing CIN2+ compared to those with negative cytology (8.5% vs. 12.3%) [83]. This method was also tested in 270 postmenopausal women with low-grade cytology results and showed specificity of 94.3% and 94.6% for the detection of CIN2+ and CIN3+, respectively [84]. In addition, in a population of 169 women with ASC-H cytology, the sensitivity and specificity for the detection of histologically confirmed HSIL in a 36-month follow-up were 95% and 72%, respectively [85].

Recently, a British cohort study compared the diagnostic accuracies of LBC, p16/Ki67 dual staining, and HPV 16/18 genotyping on a sample of 61 patients with CIN2+ compared to 279 controls with CIN1 or less [86]. The authors reported higher sensitivity for dual staining but lower specificity compared to LBC. The same research group published a subsequent analysis of the longitudinal accuracy of these modalities. These were assessed after three years in women younger than 50 years and after 5 years in women older than 50 years. Compared to LBC, dual staining had higher sensitivity and lower specificity for the detection of CIN2+ and CIN3+ [87].

Dual staining was also found to have high sensitivity and specificity values for the detection of CIN2+ and CIN3+ compared to HPV genotyping in a recent American study, indicating that this method could be used as triage for HPV-positive women [88]. p16/Ki67 dual staining was approved by the United States Food and Drug Administration (US FDA) in March 2020 for the triage of HPV-positive women, either in primary HPV screening or HPV/Pap cytology co-testing [89].

Taken together from studies conducted so far, p16/Ki-67 immunostaining combines high specificity and sensitivity for the detection of CIN2+ when used as a triage test for HPV-positive populations or for abnormal cytology [76,77,79,83,90,91,92,93]. In addition, it has the potential to decrease colposcopy referrals by up to half and is effective as a triage for women with ASC-US and LSIL [75]. Due to the fact that LSIL cytology is an indicator of productive HPV infection and HPV tests do not provide any additional information, p16/Ki-67 dual staining represents the only effective triage for women with LSIL cytology [15,75,77].

**Figure 5:**
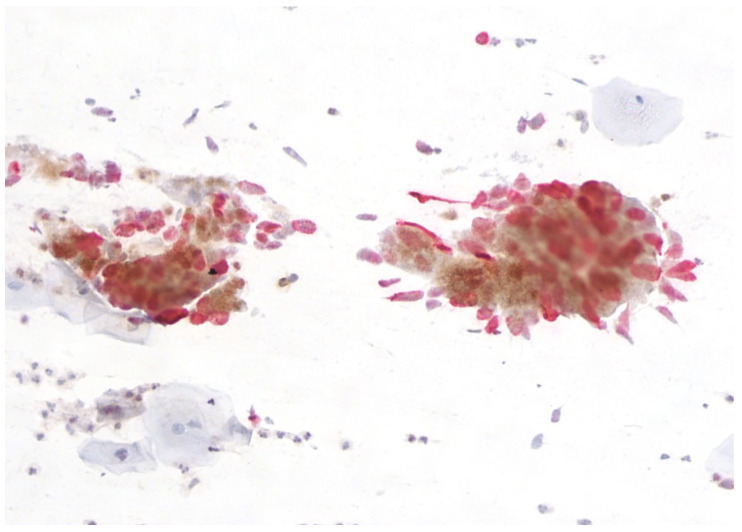
Adenocarcinoma in situ, p16/Ki67-positive reaction ×400.

There are two promising aspects of p16/Ki67 screening that need further research, namely the role of the number of p16/Ki67-positive cells in the detection of high-grade cervical precancerous lesions and the possible use of dual staining in the triage of atypical glandular cells (Figure 5).

p16/Ki67 dual staining is interpreted as positive when one or more cells with positive-p16/Ki67 immunoreactivity are found [75]. Our research group investigated the impact of the number of immunoreactive cells on the detection of CIN2+ [94]. The positive-predictive value (PPV) of detecting CIN2+ was 67.3% with five or more p16/Ki-67-positive cells, compared to 44.6% in cases with only one-positive cell [94]. However, the major limitation of our study was the very small number of included patients, as only 42 women had histologically confirmed CIN2+ [94]. Larger studies have evaluated the role of the number of positive-p16/Ki67 cells in the detection of CIN2+ and CIN3+ [82,83]. Clarke et al. reported a 49.5% 5-year risk of developing CIN2+ in cases with more than 50 dual-stained cells, compared to 12.9% in cases with only one positive cell [83]. In a Dutch retrospective study, the specificity for the detection of CIN2+ in cases with only one positive-p16/Ki67 cell was 72.8%, compared to 97.8% in cases with more than 50 positive cells [82].

The utility of p16/Ki67 dual staining for the detection of cervical adenocarcinoma was studied in a recent Japanese study [95]. The study included 142 patients, of whom 100 had cervical adenocarcinoma, 31 had benign glandular lesions, and 11 had a normal histology result. Diffuse or focal p16/Ki67 positivity was observed more often in cervical adenocarcinomas compared to normal histology, indicating that this test might be useful in triaging atypical glandular cells on cytology [95].

An important aspect of p16/Ki67 dual staining and its role in cervical cancer screening is its cost-effectiveness [96,97]. A French study evaluated the cost-effectiveness of various cervical cancer screening modalities [96]. The authors reported that the change from Pap to dual staining as a triage of positive-HPV or positive-Pap test led to a moderate increase in costs [96]. A research group from Thailand compared the cost-effectiveness of primary HPV testing with LBC as a triage and primary LBC [98]. The authors reported that primary HPV testing was less costly and more effective [98]. A subsequent analysis by the same authors compared the cost-effectiveness of p16/Ki67 dual staining as a triage test for hr-HPV-positive women with primary cytology. HPV genotyping with dual staining as a triage test was more effective than cytology but also more costly [99]. The latest study on this topic by the same research group compared the cost-effectiveness of cytology and dual staining in the setting of primary HPV testing [97]. In this study, the most cost-effective method was primary HPV genotyping with dual staining as a triage test [97].

Another important aspect of cervical cancer screening is the impact of HPV vaccination on screening strategies [100,101]. HPV vaccines have shown more than 95% protection rates against the HPV types related to the vaccine, and clinical efficacy with regards to cervical and vulvovaginal disease protection has been shown for women aged 15–45 [100]. It is expected that the positive-predictive value of low-grade cytologic abnormalities and hr-HPV testing will be reduced in vaccinated populations as HPV16/18, which is covered in all three vaccine types, is present in 70% of cervical cancers and in 30–70% of CIN2+ [101]. The positive-predictive value of the Pap test will decrease as a result of the decreased prevalence of CIN2+ among vaccinated women due to the lower prevalence of HPV16/18 infections [102]. In addition, the number of false-positive abnormal cytological results that correspond to low-risk HPV infections will increase, thus decreasing the positive-predictive value of the Pap test [102].

## 6. Conclusions

This manuscript covers all relevant research regarding the use of p16/Ki67 dual staining in cervical cancer screening. This method is promising in terms of its capability to decrease the number of unnecessary colposcopies. In addition, it could be helpful in predicting the regression or progression of CIN2 and could also be useful in triaging abnormal glandular cells.

## Figures and Tables

**Table 1:** Sensitivity and specificity values of p16/Ki67 dual staining for the detection of high-grade cervical precancerous lesions in major studies. NA—Not available.

STUDY	RISK GROUP	SENSITIVITY (%)	SPECIFICITY (%)
Ikenberg et al. (PALMS study) [76] (aged more than 30)	CIN2+	84.7	96.2
	CIN3+	87.2	95.9
Wentzensen et al. (2012) (all age groups) [80]	CIN2+	86.4	59.5
	CIN3+	93.2	46.1
Schmidt et al. (EEMAPS trial) (aged more than 18) [75]	CIN2+ (ASC-US)	90.2	80.6
	CIN3 (ASC-US)	92.2	NA
	CIN2+ (LSIL)	94.2	68.0
	CIN3 (LSIL)	95.8	NA
Petry et al. [79]	CIN2+	91.9	82.1
	CIN3+	96.4	76.9
Wentzensen et al. (2015) (all age groups) [77]	CIN2+	70.7	70.8
	CIN3+	81.3	69.6
Waldstrom et al. (all age groups) [81]	CIN2+	88.5	51.3
	CIN3+	95.7	48.2
Killeen et al. [15]	CIN2+	94.3	61.9
	CIN3+	NA	NA
Uijterwaal et al. [82]	CIN2+	68.8	72.8
	CIN3+	73.3	70.0

## Data Availability

Not applicable.
